# Hypothesis: racism is a risk factor for cerebrovascular diseases

**DOI:** 10.1590/S1516-31802006000500015

**Published:** 2006-09-07

**Authors:** Eduardo Faerstein

I very much appreciated the editorial "Stroke in Brazil: a neglected disease",^1^ because it emphasized not only the high mortality rates due to cerebrovascular diseases in Brazil but also the surprising paucity of epidemiological studies that have so far investigated their determinants in our population. The editorial appropriately draws attention to the relevance of the social determinants of these conditions. I would like to suggest that the possible direct and indirect effects of racial discrimination in Brazil should be an issue for empirical investigation as potential determinants of cerebrovascular diseases. In fact, racism constitutes an additional axis of social inequalities that generate disease and deaths in our country, and has been the subject of growing academic interest.^2^

Recently, Chor and Lima^3^ reported that, differently from whites, black men and women aged 40-69 years have mortality rates from cerebrovascular diseases that are higher than from ischemic heart disease. In addition, our exploratory analyses among participants in the Pro-Saúde study in Rio de Janeiro suggested that perceived lifetime racial discrimination can increase the risk of hypertension (one major risk factor for cerebrovascular diseases), through the intervention of and/or interaction with socioeconomic adversity. These circumstances may act directly through chronic psychosocial stress and/or through more proximal etiological mechanisms in the causal chain (e.g. related to obesity).^4^

Research on cerebrovascular diseases in Brazil should take the possible effects of racism into account at the various stages of their natural history. In multiethnic societies such as ours this may shed light both on specific societal features of ethnicity-based discrimination – a historical, ever-changing phenomenon^5^ – and on its potentially wide-ranging health consequences.
